# A Pilot Randomized Controlled Trial of Botulinum Toxin Treatment Combined with Robot-Assisted Therapy, Mirror Therapy, or Active Control Treatment in Patients with Spasticity Following Stroke

**DOI:** 10.3390/toxins14060415

**Published:** 2022-06-17

**Authors:** Jen-Wen Hung, Chu-Ling Yen, Ku-Chou Chang, Wei-Chi Chiang, I-Ching Chuang, Ya-Ping Pong, Wen-Chi Wu, Ching-Yi Wu

**Affiliations:** 1 Department of Rehabilitation, Chang Gung Memorial Hospital-Kaohsiung Medical Center, Kaohsiung 83301, Taiwan; hung0702@cgmh.org.tw (J.-W.H.); yaping0707@gmail.com (Y.-P.P.); wendy.wu224@gmail.com (W.-C.W.); 2 School of Medicine, College of Medicine, Chang Gung University, Taoyuan 33302, Taiwan; kcchang@cgmh.org.tw; 3 Department of Occupational Therapy and Graduate Institute of Behavioral Sciences, College of Medicine, Chang Gung University, Taoyuan 33302, Taiwan; weichichiang07@gmail.com (W.-C.C.); ichin0610@gmail.com (I.-C.C.); 4 School of Physical Therapy and Graduate Institute of Rehabilitation Science, College of Medicine, Chang Gung University, Taoyuan 33302, Taiwan; 5 Neuroscience Research Center, Chang Gung Memorial Hospital, Linkou, Taoyuan 33305, Taiwan; 6 Division of Cerebrovascular Diseases, Department of Neurology, Chang Gung Memorial Hospital-Kaohsiung Medical Center, Kaohsiung 83301, Taiwan; 7 Discharge Planning Service Center, Chang Gung Memorial Hospital-Kaohsiung Medical Center, Kaohsiung 83301, Taiwan; 8 Department of Senior Citizen Service Management, Yuh-Ing Junior College, Kaohsiung 80776, Taiwan; 9 Department of Occupational Therapy, I-Shou University, Kaohsiung 82445, Taiwan; 10 Department of Neurology, Chang Gung Memorial Hospital, Linkou, Taoyuan 33305, Taiwan; 11 Healthy Aging Research Center, Chang Gung University, Taoyuan 33302, Taiwan; 12 Department of Physical Medicine and Rehabilitation, Chang Gung Memorial Hospital at Linkou, Taoyuan 33305, Taiwan

**Keywords:** stroke rehabilitation, robot-assisted training, mirror therapy, conventional rehabilitation, upper extremity, motor function

## Abstract

Effects of the combined task-oriented trainings with botulinum toxin A (BoNT-A) injection on improving motor functions and reducing spasticity remains unclear. This study aims to investigate effects of 3 task-oriented trainings (robot-assisted therapy (RT), mirror therapy (MT), and active control treatment (AC)) in patients with stroke after BoNT-A injection. Thirty-seven patients with chronic spastic hemiplegic stroke were randomly assigned to receive RT, MT, or AC following BoNT-A injection over spastic upper extremity muscles. Each session of RT, MT, and AC was 75 min, 3 times weekly, for 8 weeks. Outcome measures were assessed at pretreatment, post-treatment, and 3-month follow-up, involving the Fugl-Meyer Assessment (FMA), Modified Ashworth Scale (MAS), Motor Activity Log (MAL), including amount of use (AOU) and quality of movement (QOM), and arm activity level. All 3 combined treatments improved FMA, MAS, and MAL. The AC induced a greater effect on QOM in MAL at the 3-month follow-up than RT or MT. All 3 combined trainings induced minimal effect on arm activity level. Our findings suggest that for patients with stroke who received BoNT-A injection over spastic UE muscles, the RT, MT, or AC UE training that followed was effective in improving motor functions, reducing spasticity, and enhancing daily function.

## 1. Introduction

Spasticity, a common symptom in the upper extremity (UE) after stroke, can deteriorate motor function of the paretic limbs [1]. Early studies show that focal botulinum toxin (BoNT-A) injection can reduce spasticity for 3 to 4 months by temporarily paralyzing muscle activity [2], improve passive function, such as hand hygiene, and reduce caregiver burden [3]. However, a recent meta-analysis study indicates that BoNT-A and placebo lead to similar effect on reducing spasticity [4]. This finding raises the possibility that additional intervention may be needed for patients with stroke who received BoNT-A injection. Furthermore, the effect of BoNT-A on active motor function remains uncertain [5,6,7]. It might be that spasticity is one component of the upper motor neuron syndrome; other symptoms, such as muscle weakness [1], decreased postural responses, and impaired coordination/dexterity and motor control/planning, usually coexist. These symptoms deteriorate active function [8]. Combining BoNT-A with a rehabilitation program has been suggested for the treatment of focal spasticity and dysfunction during daily or physical activity [9,10].

Task-oriented training is one evidence-based intervention to enhance motor recovery [11]. Conventional task-oriented approach involves practicing real-life functional tasks. Robot-assisted training (RT) and mirror therapy (MT), providing sensorimotor input, are two examples of task-oriented trainings. RT, with a unilateral or bilateral robot, is a massive and intensive training with a consistent manner facilitating motor skill acquisition. RT can be individualized and adjusted based on patients’ needs and offers a variety of sensorimotor feedback [12,13,14]. The Bi-Manu-Track for bilateral training produced a larger individual effect size compared with other robots with unilateral training [14]. In addition, MT, an inexpensive and promising intervention, has emerged as a feasible intervention to improve UE function [15]. During MT, a mirror is placed between two arms and the patient sees an inverse reflection while the unimpaired arm is being moved. The reflection of the unimpaired arm creates a visual illusion to enhance movement capability of the impaired arm [15,16].

The key elements leading to effective intervention for bilateral RT and MT are to induce similar neuroplastic changes, including increased cortical activation of the ipsilesional hemisphere and decreased activation of the contralesional hemisphere; that is, rebalance interhemispheric activation [15,17]. Furthermore, both could serve as priming strategies that increase effects of a subsequent motor functional training. However, MT is distinct from RT because MT provides visually guided motor imagery [18] that could be associated with the mirror neuron system [15]. RT provides kinesthesia input for motor relearning. It is unknown whether and how combining BoNT-A injection with bilateral RT vs with MT engenders differential effects on motor and related functional performance in patients with hemiplegic spasticity following stroke.

Few studies to date have investigated the effects of the combined BoNT-A and RT [19,20,21]. We recently show that the combined BoNT-A and RT is feasible and results in a positive effect on impairment and activity level in patients with stroke [21]. The other study from other research group found that the BoNT-A treatment with RT successfully induced a greater decrease in UE spasticity in patients with chronic stroke than RT alone [20].Another study found that BoNT-A combining with RT leads to a greater improvement in muscle strength than BoNT-A combining with conventional treatment with UE passive mobilization and stretching followed by exercises. However, both treatments lead to a similar amount of improvement on muscle tone reduction [19]. Furthermore, no study has combined BoNT-A with MT, although MT and RT share some similar theoretical backgrounds. Whether BoNT-A combined with RT induces greater effects than BoNT-A combined with other task-oriented interventions, such as MT, is still unknown. Therefore, the aim of this study was to investigate effects of combined treatments of (1) BoNT-A plus RT, (2) BoNT-A plus MT, or (3) BoNT-A plus dose-matched task-oriented training on motor recovery, spasticity, and daily and activity function and to compare the relative effects of those combined interventions.

## 2. Results

**Figure 1:**
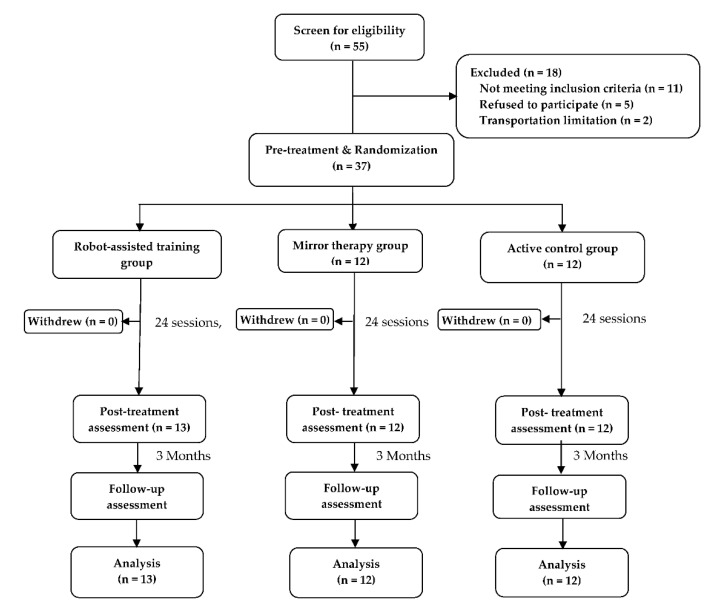
Flow diagram of the study.

We screened 55 subjects for eligibility, and 37 met the inclusion criteria and underwent randomization. Of the 37 participants, 13 were assigned into the RT group, 12 to the MT group, and 12 to the AC group (Figure 1). All participants completed the study protocol.

The BoNT-A was injected into elbow flexors, forearm pronators, wrist flexors, and/or fingers flexors. Those muscles were evenly distributed among 3 treatment groups. A similar amount of BoNT-A administered in the 3 groups (RT: 323.08 ± 117.15 IU, MT: 306.25 ± 108.20 IU, and AC: 330.00 ± 117.19 IU; *p* = 0.869).

The basic characteristics of participants’ demographic data are summarized in Table 1. The demographics of participants, including age, sex, education, brain lesion side, stroke duration, stroke type, and cognition (see MMSE) did not differ among 3 groups.

At pretreatment, the 3 groups had similar scores on the FMA, MAS, MAL, and arm activity level (*p* > 0.068 for all; Table 2). At post-treatment, all 3 groups had significant improvement in FMA, MAS, and MAL and showed similar FMA, MAS, and MAL scores (*p* > 0.073 for all; Table 3). The 3 treatments did not induce any treatment effect on the arm activity level (*p* = 0.14; Table 3). Furthermore, at the 3-month follow-up, the 3 groups indicated similar FMA and MAS scores (*p* > 0.459 for all). However, the QOM in MAL differed among the 3 groups (*p* = 0.033; large effect size, partial η^2^ = 0.187). The post hoc analysis showed that the QOM score was better in the AC group than in the MT (*p* = 0.042) and RT groups (*p* = 0.013).

## 3. Discussion

To our knowledge, this is the first study to compare the effects of RT, MT, and AC UE training in patients with chronic spastic stroke following BoNT-A injection. The findings indicate that the FMA and MAS scores in all 3 groups significantly improved after treatment and that comparable benefits were obtained across the 3 groups at post-treatment and at the 3-month follow-up. Moreover, all 3 combined treatments had beneficial effects on MAL, but the AC induced greater effect on QOM in MAL than the RT or MT at the 3-month follow-up (large effect size, partial η^2^ = 0.187). Last, all 3 combined trainings did not induce any effect on arm activity level. The findings of this study suggested that each treatment (RT, MT, and AC) is effective in treating motor function following BoNT-A in patients with stroke who also suffer from UE spasticity. Although doses and muscles for BoNT-A injection were individualized in this study, which might influence the results, there were no significant differences of muscles selected or the doses of BoNT-A injection among 3 groups (*p* = 0.869). Therefore, we believe the differences among 3 groups were not influenced by the BoNT-A injection but the combined treatments.

This study demonstrates that all 3 combined treatments induced a beneficial effect on motor recovery and spasticity assessed by FMA and MAS at post-treatment and follow-up. The finding of this study is not surprising, given that BoNT-A, RT, MT, and AC training alone have been shown to improve FMA and/or MAS [16,22,23,24]. The results of this study supported and further extended previous finding which revealed that BoNT-A and placebo lead to similar effect on reducing spasticity [4] and that the rehabilitation therapies combined with BoNT-A induces greater effects than BoNT-A alone [25]. Our study added the evidence that combination treatments may be superior to single treatment. Furthermore, the equivalent contribution to the improvements of motor impairment across 3 groups might be that they shared theoretical bases of rebalancing interhemispheric activation and priming [15,26] by RT and MT, and the motor relearning principles and neuroplasticity [22,23,27]. The specific mechanisms of kinesthetic input for motor learning and visually guided motor imagery for RT and MT, respectively, might not play the dominant role to further boost the improvement.

We suggest that therapists can treat patients with stroke who suffer from UE spasticity by using RT, MT, or AC after BoNT-A based on the condition of the clinic. The strength of RT is that RT is less labor-intensive and has the potential to save manpower by decreasing the time demands on the therapists. MT is considered a less labor-intensive and less expensive approach and can be performed in different places, such as hospitals, home, and communities. For clinics that do not have RT or MT, the AC training is also an alternative after BoNT-A.

All 3 treatments induced a beneficial effect on real-world arm use assessed by AOU and QOM of MAL, indicating that the amount of use of the affected UE and the quality of use were improved following 3 treatments. There was a trend that the improvement of QOM from pretreatment to post-treatment differed among 3 groups (*p* = 0.073; Table 3). The AC group had a greater improvement of QOM from pretreatment to the 3-month follow-up than the other 2 groups, indicating that the positive effect induced by AC group was sustained for at least 3 months after the treatment, which was unexpected. Two reasons may explain why the AC induced the greatest effects at follow-up in this study. First, it may be easier for participants in the AC group to apply the concepts learned from AC to generalize to motor and daily functions in their real life. Second, although the QOM score was similar in the 3 groups at baseline, statistically there was a tendency that the QOM score was smaller in the AC group than in the RT or MT groups. There may be much room for improvement in the AC group. However, the baseline data was used as a covariate in the statistical analysis of covariance. We therefore suggest that the influence of relatively small score in the AC group may be minimal.

Accelerometers have been developed to provide objective information of physical activity level in patients with stroke [28,29]. In this study, the RT, MT, and AC training did not influence the arm activity level assessed by accelerometers worn on both wrists of the participant. However, all 3 groups improved the MAL AOU score at post-treatment. This implies that UE training could improve the self-perceived use of affected arm, but the MAL might not be sensitive enough to reflect real arm activity level during daily life. In addition, RT and AC induced similar effect on accelerometer-assessed arm activity, inconsistent with findings of the previous study [24]. The difference between these two findings could be due to higher variability and the subject characteristics. Participants in this study had a higher degree of spasticity and lower FMA scores of the affected UE than those in the study by Liao et al. [24].

Several limitations to this study should be mentioned. First, the sample size is relatively small. A larger sample size should be included in further studies. Second, the Bi-Manu-Track we used in this study focused only on forearm and wrist movement practice. However, some patients might require distal part or hand movement practice that the Bi-Manu-Track cannot provide, possibly diminishing the RT effects. Future studies might incorporate RT training with different parts of UE depending on the individual needs. Third, doses and muscles for BoNT-A injection were individualized, which might influence the results. However, in this study, there were no significant differences of muscles selected or the doses of BoNT-A injection received among 3 groups. Therefore, we believe the differences among 3 groups were not influenced by the BoNT-A injection. Further studies could investigate whether the dose of BoNT-A injection has a significant impact on the effects of RT, MT, or AC training. Fourth, this study did not explore neural mechanisms after the combined treatments. Further studies are needed to address this issue.

In conclusion, for patients with stroke who received the BoNT-A injection over spastic UE muscles, the RT, MT, or AC training that followed were effective in reducing spasticity and improving motor function and daily function.

## 4. Materials and Methods

### 4.1. Participants

Thirty-seven patients with spastic hemiplegic stroke were recruited from the rehabilitation department of a medical center. The inclusion criteria were (1) unilateral stroke ≥6 months; (2) Modified Ashworth Scale [30] (MAS) >1 over the elbow flexor, forearm pronator, wrist flexor, and/or finger flexor muscles; (3) UE Fugl-Meyer Assessment (FMA) score of 17 to 56 [31,32]; and (4) Mini-Mental State Exam (MMSE) ≥21 [33]. The exclusion criteria were (1) pregnancy, (2) bilateral hemispheric or cerebellar lesions, (3) visual field deficits or hemineglect, (4) any contraindications for BoNT-A, (5) prior BoNT-A treatment within 4 months of enrollment, (6) joint contracture over UEs, and (7) other orthopedic or neurological diseases that would prevent adherence to the rehabilitation protocol.

To estimate the sample size, we conducted a power analysis based on our pilot study, in which the average effect size ranged from 0.4 to 0.78. An estimate of a range of 7 to 11 patients was necessary to have 80% power and a 2-sided type I error of 0.05.

### 4.2. Study Design and Paradigm

This is a randomized controlled trial study conducted in Taiwan. This study was approved by the local ethics committee and all participants signed a written informed consent form.

A computerized (block) randomization scheme was used. We stratified participants into groups based on stroke duration (<1 year or ≥1 year) and UE motor function (FMA UE score of 17–38 or 39–56). Randomization assignment was obtained through an assistant who was not involved in assessment and intervention in this study. Participants in each group were randomly assigned to the (1) RT group, (2) MT group, or (3) active control treatment (AC) group.

BoNT-A injections were administered by 1 of 2 senior rehabilitation physicians with ≥10 years of experience of BoNT-A injection. BoNT-A Purified Neurotoxin Complex (Allergan, an AbbVie Company, Irvine, CA, USA) was used, and the injection was prepared by diluting lyophilized toxin with 0.9% saline to a concentration of 50 U/mL. Location of the targeted muscle for BoNT-A was confirmed with ultrasound guidance based on the recommendation in a systematic review for BoNT-A treatment in adults with spasticity [34]. Doses and muscles selected for BoNT-A injection were individualized and recorded based on spasticity patterns and severity of spasticity.

Concurrent use of muscle relaxants, antispastic agents, and drugs having muscle relaxant properties during the study was maintained at a constant dosage throughout the study. All other routine stroke rehabilitation (e.g., physical therapy or speech therapy) that did not involve UE training proceeded as usual.

### 4.3. Intervention

After the BoNT-A injection, participants in each group received 75 min of training, 3 times weekly, for 8 consecutive weeks, under the supervision of licensed therapists. The 3 treatment groups are described as follows.

### 4.4. Robot-Assisted Training (RT) Group

**Figure 2:**
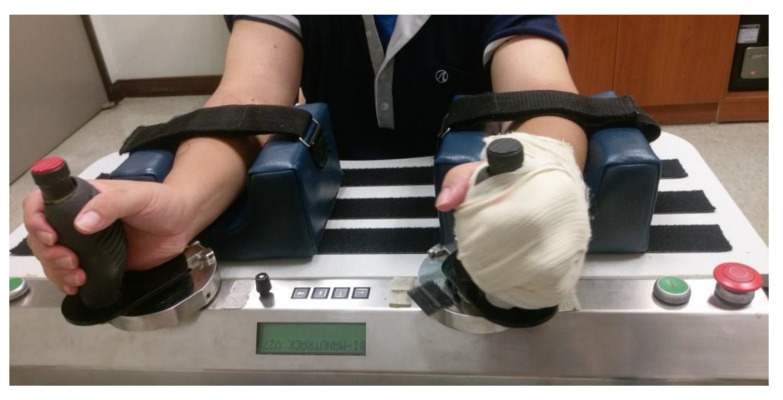
Participants in the robot-assisted therapy (RT) group received therapy with the Bi-Manu-Track (BMT; Reha-Stim Co., Berlin, Germany) robotic arm training system.

The Bi-Manu-Track (BMT; Reha-Stim Co., Berlin, Germany) robotic arm training system was used (Figure 2). Participants sat in front of a height-adjustable table, held the handles of BMT with the elbow flexed at 90°, and placed their forearms in the mid-position into the arm trough. This robotic training targeted wrist flexion-extension and forearm pronation-supination movements with 3 training modes: passive-passive (mode 1), active-passive (mode 2), and active-active (mode 3). For each movement, the participants practiced 200 repetitions (10–15 min) in mode 1, 750 repetitions (15–20 min) in mode 2, and 50 to 200 repetitions (10–15 min) in mode 3. The feedback on actions or force they exerted during practice was provided. Following 45 min of RT, participants received additional 30 min of practice in functional activities to facilitate transferring the acquired movements to daily activities. The selected functional tasks involved forearm pronation-supination or wrist flexion-extension movements, such as twisting a towel or bouncing a ball.

### 4.5. Mirror Therapy (MT) Group

**Figure 3:**
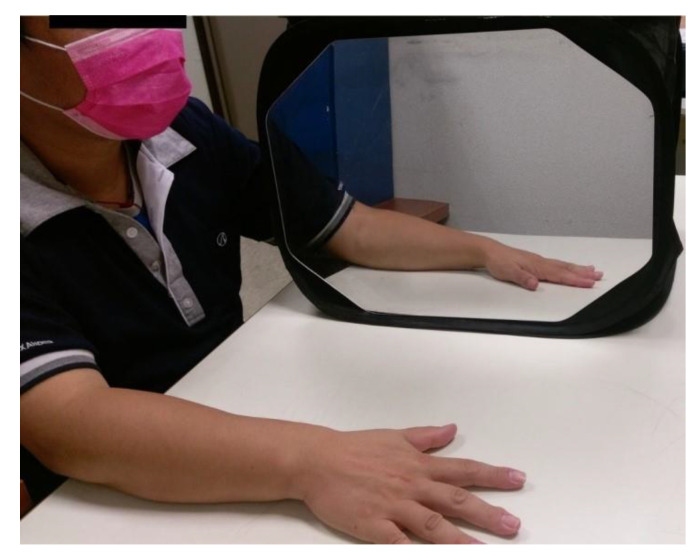
Participants in the mirror therapy (MT) group received the mirror therapy.

The MT group received 45 min of MT per session. MT included a mirror box with a mirror placed in the participant’s midsagittal plane beside the unaffected hand to block his or her view of the affected hand (Figure 3). Participants were instructed to look at the reflection of the unaffected hand in the mirror as if it were the affected hand. At the same time, they performed bilateral symmetrical movements as much as possible. The activities consisted of (1) transitive movements, such as fine motor tasks of squeezing sponges, placing pegs in holes, or flipping a card; (2) gross motor tasks of reaching out to touch a switch or keyboard; and (3) intransitive movements, including the distal part movement of the wrist, repetitive extension-flexion, or finger opponent, and the proximal part movement of forearm pronation/supination. As with participants in the RT group, participants in this group also received additional 30 min of functional practice with the same principles.

### 4.6. Active Control Treatment (AC) Group

The AC group was designed to match the RT or MT in amount of therapy hours, and these participants served as a dose-matched comparison group. Participants in this group received 45 min of conventional task-oriented approach with bilateral symmetric movement training by using affected and unaffected limbs. The movement training involved grasping, manipulating, and picking up and placing objects. After the bilateral symmetric movement training, participants received 30 min of functional practice as participants in the RT and MT group.

### 4.7. Outcome Measures

The evaluators were blind to group allocation. With the exception of the arm activity level, all assessments were performed before the BoNT-A injections, immediately after the 8-week treatment, and at the follow-up assessment 3 months after the end of the treatment. The arm activity level was only assessed before and after the treatment. Outcome measures were in accordance with the International Classification of Functioning, Disability and Health framework published by the World Health Organization World Health [35]. The UE FMA [31] and Modified Ashworth Scale (MAS) [30] were selected to assess the impairment level. The MAL [36] were selected to assess the participation level with the affected UE. Arm activity level was assessed with accelerometers.

### 4.8. Fugl-Meyer Assessment (FMA)

The UE subscale of the FMA was used to assess motor function [31]. It consists of 33 items for the reflexes and movement of shoulder, elbow, forearm, wrist, and hand, and coordination/speed. The total motor scores were divided into the proximal part (shoulder/elbow/forearm and coordination/speed) and the distal part (wrist and hand).

### 4.9. Modified Ashworth Scale (MAS)

The MAS scale, which has been shown to be reliable and valid [37], was used to evaluate spasticity over elbow flexors, forearm pronators, wrist flexors, and finger proximal interphalangeal flexor muscles of the affected limb [30].

### 4.10. Motor Activity Log (MAL)

The MAL, establishing reliability, validity, and responsiveness in patients with stroke [36], is a self-report scale to assess the real-world use of the affected arm by rating how much (amount of use scale [AOU]) and how well (quality of movement scale [QOM]) participants use their affected UE in 30 daily activities [36]. Higher scores correspond to better performance.

### 4.11. Arm Activity Level

Accelerometers were used to detect real-life arm activity level. The participants wore 2 accelerometers (Actigraph wGT3X, Pensacola, FL, USA) [38,39] on the wrist in the affected and unaffected sides for 3 consecutive days, excluding times when they were in contact with large amounts of water, such as shower, to assess arm activity level in daily life. Accelerometers worn on both wrists were able to provide valid and reliable information of real-world arm use and physical activity level in patients with stroke [28,39,40]. Accelerometer-based information has been shown to be associated with free-living physical activity and health-related quality of life in patients with stroke [29,41] and can be used as a predictor of a 90-day prognosis assessed by NIHSS [42]. The mean activity counts of the affected and unaffected arm were analyzed. 

### 4.12. Statistical Analysis

Data were analyzed with PASW statistics 18 software (IBM, Armonk, NY, USA). To examine the baseline differences among 3 groups, the *χ*^2^ was used for categorical variables and analysis of variance was used for continuous variables. To compare treatment effects between 3 groups at post-treatment and follow-up, analysis of covariance with the baseline as a covariate was used for each outcome variable. Tukey honestly significant difference post hoc tests were performed if the analyses reached significance. Partial η^2^ for effect size was also calculated (0.01 = small effect, 0.06 = medium effect, and 0.14 = large). All significant tests were two-tailed, and statistical significance was set at *α* = 0.05.

## Figures and Tables

**Table 1:** Basic characteristics of the three groups.

Variables	RT (n = 13)	MT (n = 12)	AC (n = 12)	*F*	*p* ^†^
Age (year)	47.68 ± 12.79	44.34 ± 10.05	49.71 ± 10.86	0.687	0.510
Gender (male)	10 (76.9%)	7 (58.3%)	7 (58.3%)		0.523
Education (year)	11.00 ± 3.36	13.25 ± 2.45	10.50 ± 4.50	2.072	0.142
Brain lesion (Right)	10 (76.9%)	9 (75.0%)	7 (58.3%)		0.663
Stroke duration (months)	33.38 ± 22.71	33.08 ± 16.98	38.17 ± 25.02	0.206	0.814
Stroke type					0.916
Hemorrhagic	5 (38.5%)	6 (50.0%)	5 (41.7%)		
Ischemic	8 (61.5%)	6 (50.0%)	7 (58.3%)		
MMSE	26.85 ± 2.60	28.17 ± 2.20	27.08 ± 2.31	1.064	0.356

Data are expressed as mean ± standard deviation for continuous variables and as frequency distribution (%) for categorical variables. ^†^ Categorical variables: Fisher’s exact test; continuous variables: ANOVA. Note. MMSE, Mini-Mental State Examination.

**Table 2:** Comparison the preintervention outcome measures among the three groups.

	RT (n = 13)	MT (n = 12)	AC (n = 12)	*F*	*p*
**FMA**					
UE-proximal	28.08 ± 5.30	28.58 ± 5.07	25.42 ± 7.93	0.907	0.413
UE-distal	4.85 ± 2.64	4.08 ± 4.03	4.75 ± 3.79	0.171	0.844
total	32.92 ± 7.12	32.67 ± 7.92	29.67 ± 11.15	0.510	0.605
**MAS**					
Elbow flexor	1.50 ± 0.28	1.45 ± 0.96	1.50 ± 0.36	0.019	0.981
Forearm pronator	1.69 ± 0.52	1.66 ± 0.71	1.58 ± 0.70	0.030	0.970
Wrist flexor	1.34 ± 0.59	1.50 ± 0.73	1.67 ± 0.96	0.535	0.590
Finger PIP flexor	2.46 ± 0.74	2.00 ± 2.00	2.08 ± 1.14	0.796	0.459
**MAL**					
AOU	1.47 ± 0.54	1.41 ± 0.55	1.01 ± 0.40	2.913	0.068
QOM	0.94 ± 0.53	0.88 ± 0.64	0.52 ± 0.33	2.416	0.104
**Physical Activity**					
Count in the affected side	402.17 ± 217.57	393.20 ± 225.16	606.38 ± 228.18	3.374	0.047
Count in the unaffected side	1421.48 ± 137.21	1155.32 ± 150.31	1582.54 ± 137.21	2.222	0.125

Note. FMA, Fugl-Meyer Assessment. Here, the UE-proximal score involves the shoulder, elbow, forearm, and coordination/speed subscores. The UE-distal score involves the wrist and hand subscores. MAS, Modified Ashworth Scale; PIP, proximal interphalangeal joint. MAL, Motor Activity Log; AOU, amount of use subscale; QOM, quality of movement subscale.

**Table 3:** Descriptive (**A**) and inferential statistics (**B**) of the outcome measures.

A									
	Pretreatment			Post-Treatment			Follow-Up		
	RT	MT	AC	RT	MT	AC	RT	MT	AC
**FMA**									
UE-proximal	28.08 ± 5.30	28.58 ± 5.07	25.42 ± 7.93	30.00 ± 5.60	30.08 ± 4.01	27.50 ± 8.20	29.46 ± 5.02	29.41 ± 5.16	28.41 ± 7.62
UE-distal	4.85 ± 2.64	4.08 ± 4.03	4.75 ± 3.79	6.46 ± 4.46	5.83 ± 3.27	5.41 ± 4.18	5.46 ± 3.33	5.50 ± 3.73	5.33 ± 4.03
total	32.92 ± 7.12	32.67 ± 7.92	29.67 ± 11.15	36.46 ± 8.88	35.91 ± 6.48	32.91 ± 12.07	34.92 ± 7.25	34.92 ± 8.49	33.75 ± 11.00
**MAS**									
Elbow flexor	1.50 ± 0.28	1.45 ± 0.96	1.50 ± 0.36	1.00 ± 0.74	1.00 ± 0.56	1.13 ± 0.48	1.35 ± 0.47	1.17 ± 0.44	1.29 ± 0.33
Forearm pronator	1.69 ± 0.52	1.67 ± 0.71	1.58 ± 0.70	1.08 ± 0.84	1.21 ± 0.94	1.25 ± 0.72	1.62 ± 0.58	1.46 ± 0.66	1.46 ± 0.45
Wrist flexor	1.34 ± 0.59	1.50 ± 0.73	1.67 ± 0.96	0.69 ± 0.72	1.08 ± 0.87	0.96 ± 0.50	1.12 ± 0.62	1.04 ± 0.58	1.13 ± 0.53
Finger PIP flexor	2.46 ± 0.74	2.00 ± 1.02	2.08 ± 1.14	1.85 ± 0.90	1.71 ± 0.94	1.92 ± 1.31	2.31 ± 0.83	2.21 ± 0.99	2.50 ± 0.80
**MAL**									
AOU	1.47 ± 0.54	1.41 ± 0.55	1.01 ± 0.40	1.81 ± 0.70	1.98 ± 0.94	1.54 ± 0.55	1.78 ± 0.80	1.69 ± 0.97	1.56 ± 0.58
QOM	0.94 ± 0.53	0.88 ± 0.64	0.52 ± 0.33	1.26 ± 0.74	1.35 ± 0.90	1.03 ± 0.65	1.22 ± 0.82	1.24 ± 1.00	1.08 ± 0.70
**Physical Activity**									
Count in the affected side	402.17 ± 217.57	393.20 ± 225.16	606.38 ± 228.18	398.15 ± 167.70	458.39 ± 245.80	578.12 ± 307.92	-	-	-
Count in the unaffected side	1421.48 ± 436.06	1155.32 ± 583.02	1582.54 ± 410.44	1440.98 ± 517.69	1193.43 ± 717.09	1460.86 ± 361.94	-	-	-
**B**									
		**ANCOVA**				**ANCOVA**			
		**Post-Treatment**				**Follow-Up**			
		**F**		***p* value**	**Partial η^2^**	**F**		***p* value**	**Partial η^2^**
**FMA**									
UE-proximal		0.107		0.898	0.006	0.797		0.459	0.046
UE-distal		0.690		0.509	0.040	0.477		0.625	0.028
total		0.032		0.968	0.002	0.466		0.632	0.027
**MAS**									
Elbow flexor		0.174		0.841	0.010	0.552		0.581	0.032
Forearm pronator		0.815		0.451	0.047	0.273		0.763	0.016
Wrist flexor		1.055		0.360	0.060	0.462		0.634	0.027
Finger PIP flexor		0.269		0.765	0.016	0.561		0.576	0.033
**MAL**									
AOU		1.531		0.231	0.085	1.665		0.205	0.092
QOM		2.839		0.073	0.147	3.785		0.033	0.187
**Physical Activity**									
Count in the affected side		0.634		0.537	0.041	-		-	-
Count in the unaffected side		0.443		0.646	0.029	-		-	-

Note. FMA, Fugl-Meyer Assessment. Here, the UE-proximal score involves the shoulder, elbow, forearm, and coordination/speed subscores. The UE-distal score involves the wrist and hand subscores. MAS, Modified Ashworth Scale; PIP, proximal interphalangeal joint. MAL, Motor Activity Log; AOU, amount of use subscale; QOM, quality of movement subscale.

## Data Availability

The data presented in this study are available on request from the corresponding author. The data are not publicly available due to ethical issue.
